# The radiotracer [^123^I]I-FP-CIT binds preferentially to the dopamine transporter expressed at the plasma membrane of nigrostriatal dopaminergic neurons: a new concept

**DOI:** 10.1007/s12149-025-02140-6

**Published:** 2025-12-06

**Authors:** Jan Booij, Youssef Chahid, Eric A. Reits, Ulrik Gether

**Affiliations:** 1 Department of Radiology and Nuclear Medicine, University of Amsterdam, Amsterdam UMC, Meibergdreef 9, Amsterdam, 1105 AZ The Netherlands; 2 Department of Pharmacy, University of Amsterdam, Amsterdam UMC, Amsterdam, The Netherlands; 3 Department of Medical Biology, University of Amsterdam, Amsterdam UMC, Amsterdam, The Netherlands; 4 Molecular Neuropharmacology and Genetics Laboratory, Department of Neuroscience, Faculty of Health and Medical Sciences, University of Copenhagen, Copenhagen, Denmark

**Keywords:** Dopamine transporter, [^123^I]I-FP-CIT, Internalization, Down-regulation, Plasma membrane

## Abstract

Dopamine transporter (DAT) tracers like [^123^I]I-FP-CIT are frequently used in routine practice to support the diagnosis in patients suffering from clinically uncertain parkinsonian syndromes as well as in scientific studies. The DAT is expressed not only at the plasma membrane of dopaminergic neurons, but is also trafficking within the cytoplasm. It has been well documented that the striatal [^123^I]I-FP-CIT binding is lower in disorders neuropathologically characterized by DAT loss induced by degeneration of nigrostriatal dopaminergic neurons (e.g., Parkinson’s disease). *In addition*, in studies in subjects without dopaminergic degeneration it has been suggested that *subtle* lower striatal binding can be induced by down-regulation of the DAT. However, theoretically, down-regulation can only be measured if [^123^I]I-FP-CIT binds predominantly to the DAT expressed at the plasma membrane, but not due to binding to the internalized DAT. We therefore looked into the literature to find support or opposition for this postulate. In this brief narrative review, we found indirect evidence that tracers like [^123^I]I-FP-CIT binds predominantly to the DAT expressed on plasma membrane of nigrostriatal dopaminergic neurons.

## Introduction

**Fig. 1:**
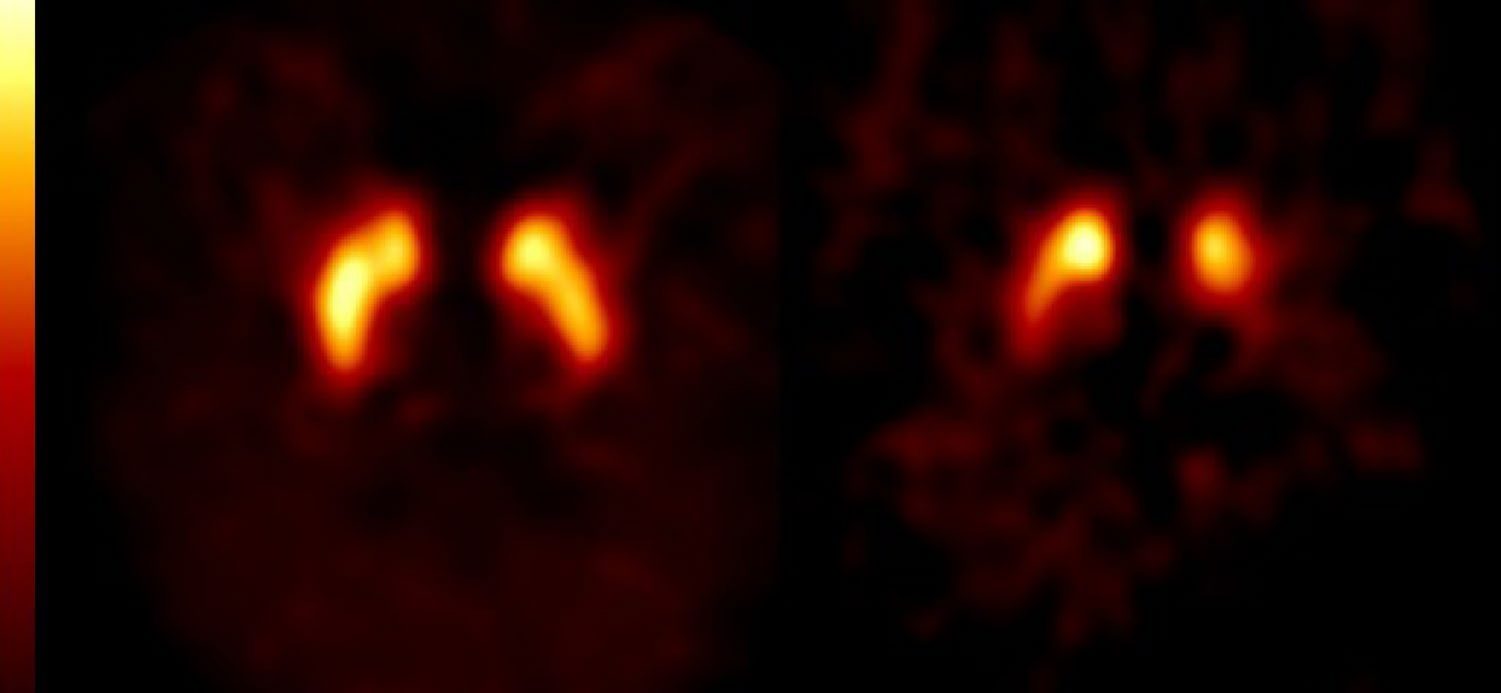
Transversal [^123^I]I-FP-CIT SPECT images at the level of the striatum. Left panel: no loss of striatal binding in a patient without nigrostriatal dopaminergic degeneration. Right panel: loss of striatal binding in a patient with nigrostriatal dopaminergic degeneration. Note the asymmetric striatal binding, and lower binding in the putamen than in the caudate nucleus, which is typical for diseases like Parkinson’s disease. Scans were acquired on a brain-dedicated SPECT system (InSpira) at the Amsterdam UMC. Reprinted from [37].

The dopamine transporter (DAT) tracer [^123^I]I-FP-CIT (or [^123^I]I-ioflupane) is used to detect or exclude nigrostriatal dopaminergic degeneration (Fig. 1) [1]. This radiotracer is derived from cocaine and binds with high-affinity to the DAT [2].

**Fig. 2:**
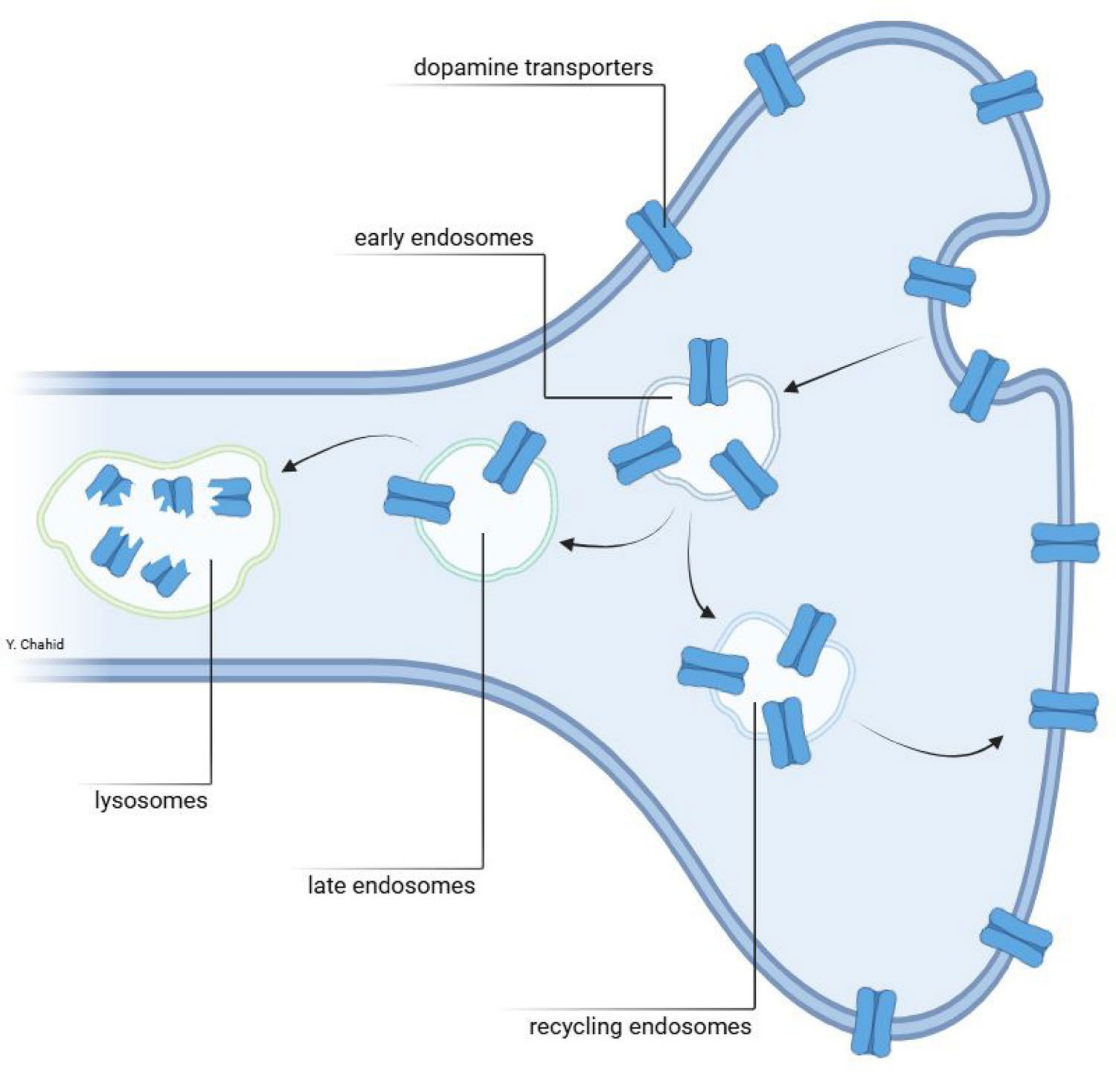
Locations of the DAT in a terminal of a dopaminergic neuron in the plasma membrane and intracellularly, and the process of trafficking of the DAT from and to the plasma membrane. The design of this figure was inspired by Fig. 2 of [4]. The DAT can internalize via endocytosis, and traffic to the so-called early endosomes. Then the majority of DATs may traffic to the Vps35 + retromer recycling endosomes, and recycle to the plasma membrane. It has been suggested that a minority of the DAT pool in early endosomes traffic to late endosomes and finally to lysosomes [4]

Interestingly, evidence has been growing that the DAT is not only expressed at the plasma membrane of terminals of dopaminergic neuron, but is also present in the cytoplasm of dopaminergic neurons [3]. More specific, the DAT is trafficking to and from the plasma membrane (Fig. 2). The DAT-1/SLC6AC gene is located on chromosome 5 and encodes the DAT, which is expressed exclusively in dopaminergic neurons [4–6]. After the DAT is synthesized in the endoplasmatic reticulum it is transported to the membrane of terminals of dopaminergic neurons. At this location, it plays a role in the regulation of the synaptic concentration of dopamine by recycling dopamine back into the neuron [3].

Importantly, the DAT itself is also subject to (re)cycling. In this regard, it is demonstrated that the DAT can internalize from the plasma membrane to the cytoplasm, more specifically to an intracellular endosomal-like pools of vesicles. Once internalized, a fraction may be targeted to degradation via late endosomes and lysosomes (Fig. 2). However, a major fraction of DAT traffics back to the plasma membrane conceivably via the retromer pathway [3].

## Why is it important to understand if DAT tracers bind in-vivo predominantly to the DAT expressed on the plasma membrane?

It may be relevant to know whether DAT tracers, like [^123^I]I-FP-CIT, bind in-vivo predominantly to the DAT located at the plasma membrane for the interpretation of DAT imaging studies. Interestingly, sometimes in studies in which [^123^I]I-FP-CIT, or similar cocaine- or methylphenidate-derived radiopharmaceuticals, were used, a lower striatal binding is (partly) interpreted as down-regulation of the DAT. For example, a [^11^C]methylphenidate PET study in Parkinson’s disease (PD) suggested that lower striatal DAT binding in early PD patients may reflect down-regulation *on top* of the loss of DAT binding induced by dopaminergic degeneration [7]. Also, Pettorruseo and co-workers showed approximately 10% lower striatal [^123^I]I-FP-CIT binding in gambling disorder [8]. They suggested that this subtle lower binding is induced by DAT down-regulation. This interpretation makes only sense if tracers like [^123^I]I-FP-CIT bind predominantly to the DAT located at the plasma membrane (Fig. 2).

## Evidence that [^123^I]I-FP-CIT binds preferentially to the plasma membrane DAT

Early human biodistribution studies showed that approximately 9% of injected [^123^I]I-FP-CIT passes the blood-brain-barrier (BBB), which indicates its lipophilicity [9]. Consequently, one might argue that this radiotracer is also lipophilic enough to pass both the membrane of dopaminergic neurons and intracellular vesicles to reach the luminal side of DAT containing vesicles. However, there is indirect evidence that [^123^I]I-FP-CIT binds preferentially to the DAT located at the plasma membrane.

**Fig. 3:**
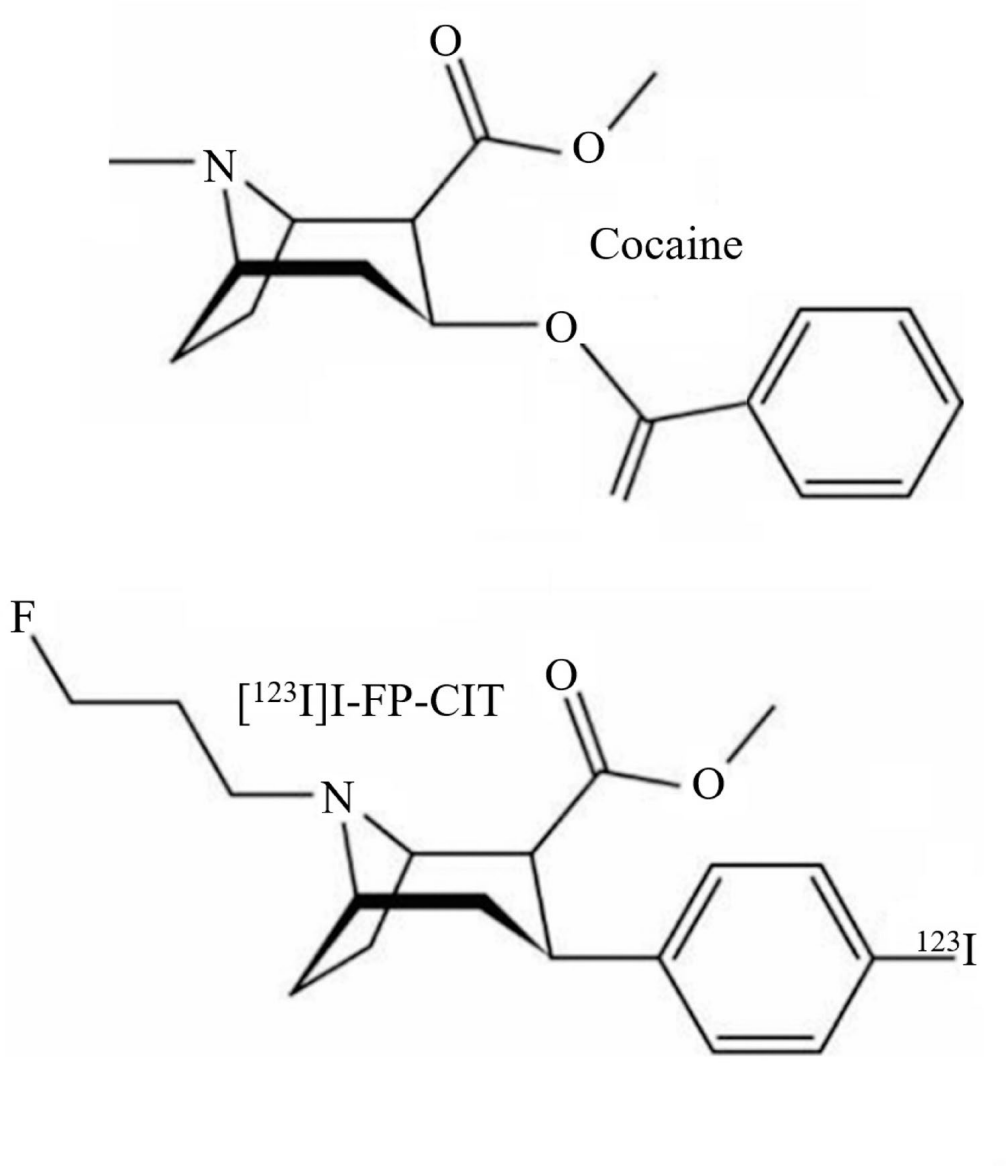
Structural formulae of cocaine (methyl (1R,2R,3 S,5 S)-3-benzoyloxy-8-methyl-8-azabicyclo[3.2.1]octane-2-carboxylate) and [^123^I]I-FP-CIT (N-ω-fluoropropyl-2β-carbomethoxy-3β-(4-iodophenyl)tropane)

**Fig. 4:**
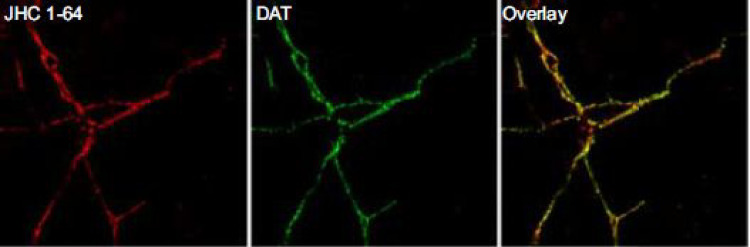
Visualization of the DAT expressed in the membrane of dopaminergic neurons in cultured postnatal midbrain cells. Left and middle panels: co-staining for the DAT with a fluorescent tracer derived from cocaine (left panel; the fluorescent tag was extended from the tropane N-position of 2-carbomethoxy-3-(3,4-dichlorophenyl)tropane using an ethylamino-linker) and rat anti-DAT antibody (middle panel). Right panel: overlay of left and middle images. Reprinted from [15]

First, dopamine itself is a substrate for the orthosteric site of the DAT. When it binds to the DAT, the plasma membrane DAT changes its conformation from the “outward facing” to “inward facing” state. This facilitates the reuptake of dopamine from the synaptic cleft [5]. Importantly, cocaine and methylphenidate are not substrates for the DAT, but DAT inhibitors. In this regard it is of interest that cocaine may bind to a central site of the DAT, and that this binding stabilizes the “outward facing state” of the DAT [10–12].Furthermore, a recent study using high-resolution microscopy demonstrated that the DAT exists in the membrane in dynamic equilibrium between an inward-facing nanodomain-localized and outward-facing unclustered configuration. Cocaine promotes dispersal of DAT from plasma membrane nanodomains to unclustered configuration stabilizing the outward-facing conformation of the DAT [13]. This mechanism may reduce the uptake of cocaine into the dopaminergic neuron. In this context, it is relevant that radiotracers like [^123^I]I-FP-CIT are chemically derived from cocaine (Fig. 3). Consistent with this notion, we previously showed in a mouse study that cocaine rapidly and extensively displaced striatal [^123^I]I-FP-CIT binding [14]. Srivastava and co-workers used another cocaine analogue, namely 2$\:{\upbeta\:}$-carbomethoxy-3$\:{\upbeta\:}$-(4-fluorophenyl)tropane (CFT), to demonstrate the outward facing state of the DAT. CFT is structurally similar to N-ω-fluoropropyl-2β-carbomethoxy-3β-(4-iodophenyl)tropane (FP-CIT) [10], which further substantiate the concept that [^123^I]I-FP-CIT may bind to the outward facing confirmation of the DAT. Also florescent radiotracers derived from cocaine bind in-vivo predominantly to the DAT located in the plasma membrane (Fig. 4) [15]. Although the underlying mechanism is unknown, it is tempting to speculative that the occupancy of a DAT at the plasma membrane, by a DAT tracer derived from cocaine, may induce or stabilize the outward conformation of the DAT, thereby preventing the internalization of that particular DAT. Consequently, the uptake of the radiotracer in a dopaminergic neuron may be prevented. Furthermore, cocaine may pass the BBB via both passive diffusion and carrier-mediated transport [16]. However, it is unlikely that dopaminergic neurons express the same carrier-mediated transport system as expressed in the BBB in their terminals, since cocaine acts pharmacologically as a typical DAT blocker and is not taken up by the DAT. Theoretically, a DAT tracer such as [^123^I]I-FP-CIT could cross the membrane of dopaminergic neurons by passive diffusion and potentially bind to internalized DAT. However, since the affinity for the DAT is very high, it is more likely that [^123^I]I-FP-CIT binds to the DAT expressed on the plasma membrane than to enter dopaminergic neurons by passive diffusion. Finally, the pH inside endosomal vesicles, that the extracellular face of the DAT will be exposed to upon internalization, is slightly acidic (pH ~ 6.0-6.5) [17]. Therefore, even if a radiotracer like [^123^I]I-FP-CIT enters a dopaminergic neuron, the more acidic intracellular environment may reduce its binding to the internalized DAT [18, 19].

Second, it is known that amphetamines are substrates for the DAT and can induce a fast down-regulation of the DAT [20]. Indeed, administration of amphetamines induces an “inward-facing” conformation of the DAT. Interestingly, already early studies in non-human primates showed that administration of amphetamine induced lower striatal [^123^I]I-2β-carbomethoxy-3-β-(4-iodophenyl)tropane (β-CIT) binding [21]. Like [^123^I]I-FP-CIT, [^123^I]I-β-CIT is derived chemically from cocaine. Although it cannot be excluded that an acute increase in synaptic dopamine levels, induced by the administration of amphetamine, may displace striatal [^123^I]I-β-CIT binding to the DAT, this alternative explanation is less likely. For example, the initiation of the use of levodopa in early PD patients did not induce significantly lower striatal [^123^I]I-FP-CIT binding [22]. Also, acute dopamine depletion or restoration in non-human primates did not influence striatal [^123^I]I-β-CIT binding [23].

Third, in a [^18^F]FP-CIT PET study, striatal DAT binding was significantly decreased in mice that lived in an environmental enrichment. This was confirmed using a surface biotinylation assay, which differentiates the DATs located at the plasma membrane from those located intracellularly [24].

## Loss of striatal [^123^I]I-FP-CIT binding in neurodegenerative disorders

It is well documented that degeneration of nigrostriatal dopaminergic cells induces loss of striatal DAT. The hallmark study of Kish and co-workers was one the first large post-mortem studies that showed the typical loss of striatal DAT in PD with a more pronounced loss in the putamen than in the caudate nucleus (Fig. 1) [25]. Later on, this finding was supported by animal studies (e.g [26]. , ; but see also [27]), DAT imaging studies [1, 7, 28, 29] as well as by studies which showed that ante-mortem loss of DAT binding was associated with dopaminergic degeneration demonstrated post-mortem [30, 31]. However, it has also been suggested that lower striatal DAT binding in early PD patients may reflect down-regulation *on top* of the loss of DAT induced by dopaminergic degeneration [7].

## Potential future studies

So, the findings of our review are consistent with, but do not definitively prove, that radiotracers like [^123^I]I-FP-CIT bind preferentially to the DAT expressed at the plasma membrane of dopaminergic neurons. To come up with direct evidence, it would be helpful to use techniques to detect the in-vivo binding of the radiotracer *itself* at the subcellular level. As far as we know, however, such techniques are not available yet.

The second best option would be to add a florescent tag to the FP-CIT molecule and visualize the distribution using confocal imaging in mice, or use click chemistry to label bound FP-CIT and visualize its distribution using fluorescence or electron microscopy (Fig. 4) [15]. The disadvantage of this approach is that the physicochemical differences between the fluorescent cocaine analog and the native [^123^I]I-FP-CIT may affect binding to the DAT. Their molecular size, polarity, and lipophilicity likely differ, which may influence membrane permeability and binding properties. Also, results may be species dependent [32]. Another option would be to perform studies in genetically modified animals. For example, Skinbjerg and co-workers demonstrated that dopamine D_2_ receptor PET tracers preferentially bind to the dopamine D_2_ receptor located at the plasma membrane, but not to internalized D_2_ receptors, using a mice knockout model. These knockout mice are incapable to internalize D_2_ [33]. However, to our knowledge there is no knockout model which is incapable of internalizing the DAT. Although results obtained in knockout animals are relevant to understand mechanism of binding of radiotracers, the results may be species dependent. In addition, future in-vitro experiments on cell lines expressing the DAT at the plasma membrane as well as intracellularly may be of interest. Similar to studies evaluating potential PET tracers for the somatostatin receptor subtype 2, cell lines expressing the DAT may be incubated with [^123^I]I-FP-CIT. After incubation, membrane-bound radioactivity can be removed, and the cells subsequently lysed to assess any internalized radioactivity [34]. A limitation of this approach is that the translation to the in-vivo situation in humans is not straightforward. For example, the incubation conditions may not reflect the low occupancy of striatal DATs by [^123^I]I-FP-CIT in-vivo. It is likely that less than 1% of DATs are occupied in a typical [^123^I]I-FP-CIT SPECT study in humans [26, 35]. Also, such cell lines may be used to assess if the affinity of [^123^I]I-FP-CIT for the DAT expressed in the membrane may differ from that for DATs that are internalized, as previously investigated for dopamine D_2_ receptor tracers [36]. Finally, it might be helpful to get more indirect evidence on this topic. In this regard, it has been suggested that under basal conditions, DAT internalization is limited by an “endocytic brake” [3]. This brake can be released by the administration of drugs. For example, the dopamine D_2_ agonist sumanirole may induce an increase in plasma membrane DAT binding [3]. In future studies, such biotinylation studies may complement [^123^I]I-FP-CIT SPECT studies in small animals to test the hypothesis that sumarirole administration induces a higher striatal [^123^I]I-FP-CIT binding in-vivo.

## Conclusions

We raise the concept that radiotracers like [^123^I]I-FP-CIT bind predominantly to the DAT expressed on the plasma membrane of dopaminergic neurons. However, it is relevant that this concept is based on indirect evidence, and should therefore be interpreted with caution.
